# Complement gene expression and regulation in mouse retina and retinal pigment epithelium/choroid

**Published:** 2011-06-14

**Authors:** Chang Luo, Mei Chen, Heping Xu

**Affiliations:** Centre for Vision and Vascular Science, School of Medicine, Dentistry and Biomedical Sciences, Queen’s University Belfast, Belfast, UK

## Abstract

**Purpose:**

To understand the expression of genes involved in different complement pathways in the retina and retinal pigment epithelium (RPE)/choroid under physiologic conditions and how their expression is regulated by inflammatory cytokines.

**Methods:**

The expression of complement components of the classical pathway (CP), mannose-binding lectin (MBL) pathway, alternative pathway (AP), and terminal pathway in the retina and RPE/choroid was determined by conventional reverse transcription polymerase chain reaction (RT–PCR). The effect of inflammatory cytokines, tumor necrosis factor-alpha (TNF-α, 20 ng/ml), interleukin (IL)-6 (10 ng/ml), interferon-gamma (IFN-γ, 100 ng/ml) or lipopolysaccharides (LPS, 1 μg/ml) on the expression of these complement component genes was tested in vitro in primary cultured RPE cells and a microglial cell line (BV2 cells) and quantified by real-time RT–PCR.

**Results:**

In the CP, complements C1qb, C1r, C1s, C2, and C4 were constitutively expressed by retina and RPE/choroid. Complement factor H and factor B of the AP as well as C3 were also detected in the retinal and RPE/choroidal tissues. In the MBL pathway, low levels of mannose-binding lectin (MBL)-associated serine protease (MASP)-1 in the retina and RPE/choroid and MASP2L in the retina were detected. Other components, including mannose-binding lectin 1 (MBL1), mannose-binding lectin 2 (MBL2), complement factor I (CFI), complement component 5 (C5) and complement factor H-related protein 1 (CFHR1), were not detected in either the retina or the RPE/choroid. The expression of CP- and AP-complement component genes in RPE and microglial cells was upregulated by interferon (IFN)-γ treatment. Treatment with TNF-α selectively upregulated the expression of C1s and C3 genes but downregulated complement factor H gene expression in RPE and microglial cells. The expression of genes involved in the MBL pathway was not affected by the inflammatory cytokines tested in this study.

**Conclusions:**

Retina and RPE/choroid express a variety of complement components that are involved mainly in the CP and AP. RPE and microglial cells are the main sources of retinal complement gene expression. Retinal complement gene expression is regulated by inflammatory cytokines, such as IFN-γ and TNF-α.

## Introduction

The retina is segregated from the circulation by the blood retinal barrier and is considered to be an immune “privileged” tissue. It is, however, still under the surveillance of the innate immune system by specialized myeloid-derived cells, including microglia [1,2], perivascular macrophages [1,2] and a novel population of dendritic cells that express 33D1 and major histocompatibility complex class II molecules [3]. Under normal physiologic conditions, these cells are in a “quiescent” state but are able to sense exogenous and endogenous danger stimuli. Once engaged with these signals, the cells are activated, resulting in retinal inflammation [4]. In addition to myeloid-derived cells, compelling evidence suggests that proteins of the complement system also exist in the retina [5-8], which together comprise the retinal innate immune system.

Activation of the retinal innate immune system (e.g., microglia and the complement system) has been shown to be involved not only in retinal diseases with overt inflammation, such as uveoretinitis [9-11], but also in a variety of retinal degenerative diseases, including age-related macular degeneration [5,12-14], diabetic retinopathy [15,16], and glaucomatous retinopathy [17]. Modulating retinal immune activation provides a novel therapeutic approach to control these pathological conditions. The roles of retinal microglia/macrophages in various retinal diseases have been extensively studied [4]. However, our knowledge on the retinal complement system, such as which complement components are expressed in the retina and how their expression is regulated, remains limited.

The complement system can be activated through the classical pathway (CP), the alternative pathway (AP), and the mannose-binding lectin (MBL) pathway. In addition, complement can also be activated independently of complement component 3 (C3) by a direct action of thrombin on the C5 convertase [18]. All pathways of the complement system lead to formation of the cytolytic membrane attack complex. The complement system consists of over 25 proteins and protein fragments, and different activation pathways require contributions from different proteins. Complement proteins are generally synthesized in the liver by hepatocytes and released as inactive precursors (proproteins) into the blood for tissue distribution. Under pathological conditions in which the blood retinal barrier breaks down, complement proteins may leak into retinal tissue, resulting in local complement activation. However, recent work from our group and others has shown that certain complement components, such as complement component 1q (C1q) [6,19], C3 [6,19], and complement regulatory factors (e.g., factor H (CFH) [6,20], factor B (CFB) [6,21], CD59 [7,22]), can also be synthesized locally by retinal cells, suggesting that de novo complement production may contribute to its activation in this setting.

The present study was undertaken to investigate the gene expression of key proteins involved in the CP, AP, and MBL pathways of the complement system in the retina and retinal pigment epithelium (RPE)/choroid under physiologic conditions. We also aimed to understand the sources of these complement proteins and how their expression is regulated by inflammatory cytokines.

## Methods

### Animals

C57BL/6 mice (8–12 weeks of age) were purchased from Harlan Laboratories Ltd. (Oxon, UK) and maintained in the Biologic Resource Unit at Queen’s University Belfast. Postnatal day 14 C57BL/6 mice were supplied by the Biologic Resource Unit at Queen’s University Belfast. All mice were housed in a standard experimental room and exposed to a 12 h:12 h light–dark cycle. The study was conducted in compliance with the Association for Research in Vision and Ophthalmology statement for the Use of Animals in Ophthalmic and Vision Research, and all animal experiments were performed under the regulations of the UK Animal License Act 1986.

### Sample collection

Retinal and RPE/choroidal tissues were collected from adult (8–12 weeks of age) C57BL/6 female mice. Mice were sacrificed with CO_2_ inhalation; eyes were carefully enucleated and further dissected under a surgical microscope. The anterior segment of the eye including the cornea, iris, and the lens were removed; retinas were peeled off from RPE/choroid. Retina, RPE/choroid, and liver tissue samples were snap frozen in liquid nitrogen for further RNA extraction.

### Cell cultures

Primary mouse RPE cells were isolated and cultured from C57BL/6 mice using a previously described method with slight modifications [23,24]. In brief, after removing the anterior segment of the eye and the lens, the eyecups were incubated with 0.5% (weight [w]/volume [v]) trypsin-EDTA (ICN Flow, Irvine, UK) at 37 °C for 1 h. The cells were then harvested by gentle aspiration, and single-cell suspensions were seeded into culture dishes with Dulbecco’s Modification of Eagle’s Medium (DMEM; Gibco BRL, Paisley, UK) containing 10% (v/v) fetal calf serum (FCS; Sigma, Cambridge, UK) and 100 µg/ml primocin (Invivogen, San Diego, CA). Upon reaching confluence, RPE cells were subcultured (1:3 ratio) and their phenotype was confirmed by RPE65 and cytokeratin staining [25]. The third passage of RPE cells was used for further experiments. In addition, a novel mouse RPE cell line, B6-RPE07, established in our laboratory was also used in this study and maintained in DMEM with 10% FCS [25].

Retinal microglial cells were cultured from day 14 postnatal C57BL/6 mice using a previously described method [26]. Briefly, retinas were cut into small pieces and incubated with 1 mg/ml type I collagenase (Sigma-Aldrich, Dorset, UK), 0.3 mg/ml DNase I (Sigma-Aldrich), and 0.2 mg/ml hyaluronidase (Sigma-Aldrich) at 37 °C for 20 min before the cell suspension was filtered through a 100-µm cell strainer (BD Biosciences, Oxford, UK). Cells were then cultured for 10 days in 24-well plates containing DMEM/F12 with 10% FCS, supplemented with 20% L929-conditioned medium containing M-CSF [27]. Immunocytochemistry confirmed that approximately 98% of the cells were CD11b^+^ and approximately 92% were F4/80^+^.

Brain microglia were cultured from mouse brain tissue using the same method described above for retinal microglial cells. A mouse microglial cell line, BV-2 [28], was cultured in DMEM with 10% FCS and 100 μg/ml primocin and incubated in 5% CO_2_ at 37 °C.

### Pro-inflammatory cytokine treatment

The third passage mouse RPE cells or BV-2 cells were cultured in 12-well plates at a density of 3×10^4^ cells/well. After reaching confluence, cells were washed with PBS (137 mM NaCl, 2.7 mM KCl, 8 mM Na_2_HPO_4_ and 1.46 mM KH_2_PO_4_ in distilled water, Ca^2+^ and Mg^2+^ free) and treated with TNF-α (20 ng/ml), IL-6 (10 ng/ml), IFN-γ (100 ng/ml; R&D Systems, Abingdon, UK), or LPS (1 µg/ml; Sigma-Aldrich) for 20 h. The cells were then collected for total RNA extraction. Triplicate samples were used for each treatment. The concentration of cytokines used in this experiment was based on our previous experience with complement gene expression in RPE cells [20,21].

### RNA isolation and reverse transcription

Total RNA was extracted from tissues or cultured cells using the RNeasy mini kit (Qiagen Ltd., West Sussex, UK) according to the manufacturer’s instructions. An RNase-free DNase Set (Qiagen Ltd.) was used for optional DNase treatment. The quantity and quality of the RNA was determined using a NanoDrop ND-1000 spectrophotometer (NanoDrop Technologies, Wilmington, DE). First-strand cDNA synthesis was performed by a reaction of 2.5 µg of total RNA with a random primer, using the SuperScript™ II Reverse Transcriptase kit (Invitrogen, Paisley, UK).

### Conventional RT–PCR

Conventional RT–PCR was performed in the retina, RPE/choroidal tissue, or cultured cells, using the promega GoTaq® Flexi DNA Polymerase kit (Promega UK, Southampton, UK). The PCR for selected genes was performed for 40 cycles, and PCR products were observed by agarose gel electrophoresis. Amplifications of complement components in the liver acted as positive controls. The primers were designed using the NCBI Primer-BLAST and are listed in Table 1.

**Table 1:** List of primers used in PCR studies.

**Entrez ID**	**Gene symbol**	**Primers (5′-3′)**	**Product size (bp)**
12260	*C1qb*	F: ATAAAGGGGGAGAAAGGGCT R: CGTTGCGTGGCTCATAGTT	301
50909	*C1r*	F: GCCATGCCCAGGTGCAAGATCAA R: TGGCTGGCTGCCCTCTGATG	313
50908	*C1s*	F: TGGACAGTGGAGCAACTCCGGT R: GGTGGGTACTCCACAGGCTGGAA	256
12263	*C2*	F: CTCATCCGCGTTTACTCCAT R: TGTTCTGTTCGATGCTCAGG	178
12266	*C3*	F: AGCAGGTCATCAAGTCAGGC R: GATGTAGCTGGTGTTGGGCT	167
12268	*C4*	F: ACCCCCTAAATAACCTGG R: CCTCATGTATCCTTTTTGGA	320
15139	*C5*	F: AGGGTACTTTGCCTGCTGAA R: TGTGAAGGTGCTCTTGGATG	173
12630	*CFI*	F: TTTCCCAACGAGTCTGTCCT R: TGCAGTCCACCTCACCATTA	194
50702	*CFHR1*	F: TTCTGGACTCGCATCACTTG R: AGCCTTGATTGCAGACCACT	157
14962	*CFB*	F: CTCCTCTGGAGGTGTGAGCG R: GGTCGTGGGCAGCGTATTG	264
12628	*CFH*	F: CGTGAATGTGGTGCAGATGGG R: AGAATTTCCACACATCGTGGCT	248
17194	*MBL1*	F: AGGGAGAACCAGGTCAAGGGCT R: ACTGCCCTTCAGTCGCCTCGT	414
17195	*MBL2*	F: CCCTGCCTGCAGTGACACCA R: AGCACCCAGTTTCTCAGGGCT	443
17174	*MASP1*	F: AGGACCTGCCGAGTGGAATG R: TCTCCACAGAAGGGACCCCA	251
17175	MASP2 isoform 1 (*MASP2L*)	F: CCTGCAGAGCGGGCTACGTT R: AGGGCCGTGCTGTGCTTGTG	402
17175	MASP2 isoform 2 (*MASP2S*)	F: ACTGACTGCACCCCCTGGCT R: GGGCTGTTGCTGAGGGAGGT	435
11461	*ACTB*	F: CCTTCCTTCTTGGGTATG R: TGTAAAACGCAGCTCAGTAA	367

F: forward; R: reverse

### Real-time reverse transcription (RT)-PCR

Real-time RT–PCR was performed on a total of 20 µl of mixture solution in 96-well plates using the LightCycler 480 system (Roche Applied Science, Mannheim, Germany). Each 20 µl of reaction mixture contained 10 µl of LightCycler 480 SYBR Green Master (Roche Diagnostics GmbH, Mannheim, Germany), 0.5µM primers, and diluted cDNA. Real-time PCR quantifications were run in triplicate for each sample, the average was determined, and PCR products were quantified using the LightCylcer 480 software. Melting curve and gel electrophoretic analyses were used to determine amplification homogeneity and data quality. Expression levels were normalized to β-actin. Gene-fold changes of treated versus untreated control groups were calculated by dividing the gene expression levels of corresponding samples.

### Statistical analysis

All data were presented as means±standard error of the mean. The difference between the treated group and control group was compared using an unpaired Student *t* test, with p<0.05 considered to be statistically significant.

## Results

### Complement gene expression in mouse retina and retinal pigment epithelium/choroid

#### Classical pathway

**Figure 1:**
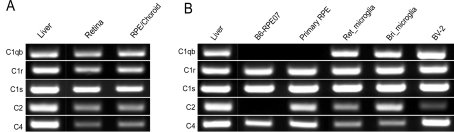
The expression of selected classical pathway-related complement genes. The expression of *C1qb*, *C1r*, *C1s*, *C2*, and *C4* in retina and retinal pigment epithelium (RPE)/choroid (**A**) and primary cultured RPE cells, B6-RPE07 cell line, primary cultured retinal microglia (Ret_microglia), brain microglia (Bri_microglia), and BV-2 cells (**B**) were analyzed by reverse transcriptase-PCR. Total RNA from mouse liver tissue was used as a positive control.

We examined the expression of complement C1 subunits (*C1qb*, *C1r*, *C1s*) and complement components (*C2* and *C4*) genes in this pathway. All of these genes were found to be expressed in mouse retina and RPE/choroid (Figure 1A). To further understand which cells might be the sources of the CP-complement gene expression in retina and RPE/choroid, we then examined microglia and RPE cells. Under normal culture conditions, primary microglial cells (originated from either mouse retina or brain) and the mouse microglial cell line (BV-2) expressed all five CP-complement genes (Figure 1B), suggesting that microglia may be one of the main sources of CP complement gene expression in the retina. Primary cultured RPE cells expressed *C1r*, *C1s*, *C2*, and *C4* but not *C1qb* (Figure 1B), whereas cells of the mouse RPE cell line B6-RPE07 only expressed *C1r*, *C1s*, and *C4*. These results suggest that *C1qb* gene expression detected in RPE/choroidal tissue might be attributed from choroidal macrophage/dendritic cells and that the B6-RPE07 cells may have lost *C2* gene expression during prolonged in vitro culture.

#### Mannose-binding lectin (MBL) pathway

**Figure 2:**
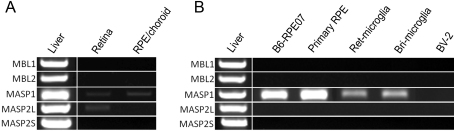
The expression of selected mannose binding lectin (MBL) pathway-related complement genes. The expression of MBL1, *MBL2*, *MASP1*, *MSAP2L*, and *MASP2S* in retina and RPE/choroid (**A**) and primary cultured retinal pigment epithelium (RPE) cells, B6-RPE07 cell line, primary cultured retinal microglia (Ret_microglia), brain microglia (Bri_microglia), and BV-2 cells (**B**) were analyzed by reverse transcriptase-PCR. Total RNA from mouse liver tissue was used as a positive control.

Five genes of this pathway, including two receptors (*MBL1* and *MBL2*) and three MBL-associated serine protease (*MASP*) genes (*MASP-1*, *MASP2L*, and *MASP2S*), were examined. Weak expressions of *MASP1* in the retina and RPE/choroid and of *MASP2L* in the retina were detected (Figure 2A). Neither the retina nor the RPE/choroid expressed detectable levels of *MBL-1*, *MBL-2*, and *MASP2S* genes (Figure 2A). Under normal culture conditions, the expression of the *MASP1* gene was detected in RPE and primary cultured microglial cells (Figure 2B). Although a low level of *MASP2L* was detected in the retina, microglial cultures did not express detectable levels of this gene (Figure 2B).

#### Alternative pathway

**Figure 3:**
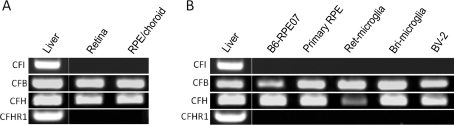
The expression of selected alternative pathway-related complement genes. The expression of *CFI*, *CFH*, *CFB*, and *CFHR1* in retina and retinal pigment epithelium (RPE)/choroid (**A**) and primary cultured RPE cells, B6-RPE07 cell line, primary cultured retinal microglia (Ret_microglia), brain microglia (Bri_microglia), and BV-2 cells (**B**) were analyzed by reverse transcriptase-PCR. Total RNA from mouse liver tissue was used as a positive control. *CFH*, complement factor H; *CFB*, complement factor B; *CFI*, complement factor I *CFHR1*, complement factor H-related protein 1.

In the AP, *CFH* and *CFB* are strongly expressed in both retina and the RPE/choroid (Figure 3A). In contrast, neither the retina nor the RPE/choroid expressed detectable levels of CFH-related protein-1 (*CFHR1*) and complement factor I (*CFI*) genes (Figure 3A).

We have shown previously that CFH and CFB are produced by RPE cells [20,21], and this was further confirmed in this study. Both primary cultured RPE cells and the mouse RPE cell line B6-RPE07 expressed high levels of CFH and CFB (Figure 3B). In addition, primary cultured microglial cells and BV-2 cells also expressed high levels of CFH and CFB (Figure 3B). Neither RPE cells nor microglial cells express detectable levels of CFHR1 and CFI under normal culture conditions (Figure 3B).

#### C3 and C5 genes

**Figure 4:**
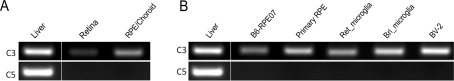
The expression of complement C3 and C5 genes. The expression of *C3* and *C5* in retina and retinal pigment epithelium (RPE)/choroid (**A**) and primary cultured RPE cells, B6-RPE07 cell line, primary cultured retinal microglia (Ret_microglia), brain microglia (Bri_microglia), and BV-2 cells (**B**) were analyzed by reverse transcriptase-PCR. Total RNA from mouse liver tissue was used as a positive control.

Complement C3 and C5 are essential for the full activation of the complement system in all three pathways. Interestingly, *C3* but not *C5* is expressed in the retinal and RPE/choroidal tissues (Figure 4A) as well as in cultured RPE and microglial cells (Figure 4B).

### The effect of inflammatory cytokines on complement gene expression

Having shown that retinal and RPE/choroidal tissues express genes involved in the CP, AP, and MBL pathway under normal physiologic conditions and that microglial and RPE cells are the major sources of complement gene expression in the retina and RPE/choroid, we then sought to understand how expression of these genes may be affected under inflammatory conditions. Cultured mouse RPE and microglial cells were treated with three inflammatory cytokines: TNF-α, IFN-γ, and IL-6. In addition, the endotoxin LPS was used as a positive control for inflammatory stimuli. Complement gene expression was quantitatively analyzed by real-time RT–PCR. For microglia we used a stabilized cell line BV-2. For RPE cells we used primary cultured mouse RPE cells as the mouse RPE cell line B6-RPE07 has been shown to have lost the expression of some of the complement genes (e.g., *C2*, Figure 1).

### Classical pathway complement gene expression

**Figure 5:**
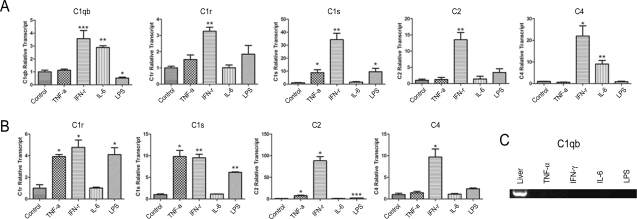
The effect of inflammatory cytokine on classical pathway-related complement gene expression in BV-2 and retinal pigment epithelium cells. BV-2 cells (**A**) and primary cultured mouse RPE cells (**B**, **C**) were treated with tumor necrosis factor (TNF)-α (20 ng/ml), interleukin (IL)-6 (10 ng/ml), interferon (IFN)-γ (100 ng/ml), or lipopolysaccharides (LPS; 1µg/ml) for 20 h. Cells were then collected for real-time RT–PCR (**A**, **B**) or conventional RT–PCR (**C**). Mouse liver RNA was used as a positive control in **C**. n=3 in each group. *p<0.05; **p<0.01; ***p<0.001 as compared to untreated control group using unpaired Student's *t*-test. Experiments were repeated twice.

Among the three pro-inflammatory cytokines, IFN-γ strongly upregulated expression of all CP-related genes in both microglial cells (Figure 5A) and RPE cells (Figure 5B). The highest upregulation was observed in the *C2* gene in RPE cells, which showed over an 80 fold increase upon 100 ng/ml IFN-γ treatment (Figure 5B). TNF-α significantly increased *C1s* gene expression in BV-2 cells (Figure 5A) and *C1r*, *C1s*, and *C2* gene expression in RPE cells (Figure 5B). Apart from *C1qb*, which was upregulated by IL-6 in BV-2 cells, all other CP-related genes were not affected by IL-6 (Figure 5). Treatment with LPS upregulated the expression of *C1s* in BV-2 cells (Figure 5A) and *C1s* and *C1r* genes in RPE cells (Figure 5B).

*C1qb* gene expression was not detected in RPE cells under normal culture conditions (Figure 1B) and was not induced by treatment with a range of different pro-inflammatory cytokines or LPS (Figure 5C).

### MBL pathway related complement gene expression

**Figure 6:**
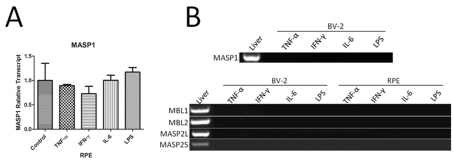
The effect of inflammatory cytokine on mannose binding lectin pathway-related complement gene expression in BV-2 and retinal pigment epithelium cells. Primary RPE cells and BV-2 cells were treated with pro-inflammatory cytokines tumor necrosis factor (TNF)-α (20 ng/ml), interferon (IFN)-γ (100 ng/ml), interleukin (IL)-6 (10 ng/ml), and lipopolysaccharides (LPS; 1 µg/ml) for 20 h. Cells were then collected for real-time RT–PCR (**A**) or conventional RT–PCR (**B**, **C**). Mouse liver RNA was used as a positive control in **B** and **C**. n=3 in each group. Experiments were repeated twice.

In the MBL pathway, MASP1 but not MBL1, MBL2, or MASP2S was detected in cultured RPE cells (Figure 2B). The expression of MBL-related genes in RPE cells was not affected by either LPS or any of the pro-inflammatory cytokine treatments (Figure 6A,B). BV-2 cells did not express any of the MBL pathway-related genes under normal culture conditions (Figure 2B), and treatment of the cells with pro-inflammatory cytokines or LPS also did not induce the expression of any of these genes (Figure 6B).

### Alternative pathway (AP*)* complement gene expression

**Figure 7:**
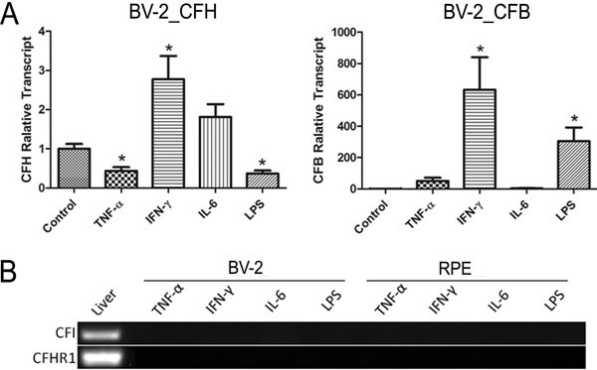
The effect of inflammatory cytokine on alternative pathway-related complement gene expression in BV-2 and retinal pigment epithelium cells. Primary RPE cells and BV-2 cells were treated with pro-inflammatory cytokines TNF-α (20 ng/ml), interferon (IFN)-γ (100 ng/ml), interleukin (IL)-6 (10 ng/ml), and lipopolysaccharides (LPS; 1 µg/ml) for 20 h. Cells were then collected for real-time RT–PCR (**A**) or conventional RT–PCR (**B**). Mouse liver RNA was used as a positive control in **B**. **A**: The effect of inflammatory cytokines on *CFH* and *CFB* genes expression in BV-2 cells. **B**: The effect of inflammatory cytokines on *CFI* and *CFHR1* gene expression in BV-2 and RPE cells. n=3 in each group. *p<0.05 when compared to untreated control group using unpaired Student's *t* test. Experiments were repeated twice.

We have previously shown that inflammatory cytokines (TNF-α, IFN-γ, and IL-6) downregulate *CFH* gene [20] but upregulate *CFB* gene [21] expression in RPE cells. In the present study in BV-2 cells, *CFH* expression was downregulated by treatment with TNF-α and LPS but upregulated by IFN-γ (Figure 7A). The expression of *CFB* in BV-2 cells was not affected by TNF-α but was massively upregulated by IFN-γ and LPS (Figure 7A).

*CFI* and *CFHR1* genes were not expressed in microglia or RPE cells under normal culture conditions (Figure 3). Treatment of the cells with TNF-α, IFN-γ, IL-6, or LPS did not induce the expression of these genes (Figure 7B).

### Complement C3 and C5 gene expression

**Figure 8:**
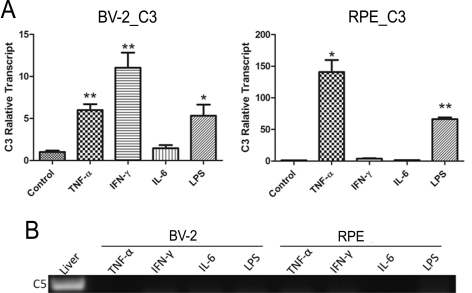
The effect of inflammatory cytokine on complement *C3* and *C5* gene expression in BV-2 and retinal pigment epithelium cells. Primary RPE cells and BV-2 cells were treated with pro-inflammatory cytokines tumor necrosis factor (TNF)-α (20 ng/ml), interferon (IFN)-γ (100 ng/ml), interleukin (IL)-6 (10 ng/ml), and lipopolysaccharides (LPS;1 µg/ml) for 20 h. Cells were then collected for real-time RT–PCR analysis of *C3* gene expression (**A**) or conventional RT–PCR analysis of *C5* gene expression (**B**). Mouse liver RNA was used as a positive control in **B.** n=3 in each group. *p<0.05; **p<0.01 when compared to untreated controls using unpaired Student's *t* test. Experiments were repeated twice.

Complement C3 was constitutively expressed by microglial and RPE cells (Figure 3A), and the levels were further enhanced by TNF-α and LPS treatment (Figure 8A). IFN-γ also increased *C3* gene expression in BV-2 cells (Figure 8A). *C3* gene expression in RPE cells was not affected by IFN-γ (Figure 8A).

The *C5* gene was not expressed in microglia or RPE cells under normal culture conditions (Figure 4), and treatment of cells with inflammatory cytokines or LPS did not induce its expression (Figure 8B).

## Discussion

Here, we show that murine retina and RPE/choroid express key complement components involved in the CP and AP and that retinal microglia and RPE cells are the major sources of complement gene expression. Furthermore, we demonstrated that the expression of retinal complement protein/regulatory factors is regulated by inflammatory cytokines, such as IFN-γ and TNF-α. In relation to the MBL pathway, only a low level of *MASP2L* expression in retina and *MASP1* expression in retinal and RPE/choroidal tissues was detected, and their expression in RPE or microglial cells was not affected by inflammatory cytokines. Our results suggest that the CP and AP but not the MBL pathway of the complement system may play an important role in retinal immunity.

Our observation of the complement gene expression profile on murine retina and RPE/choroid is in agreement with a recent study in man by Anderson et al. [6] in which the authors reported that human retina, choroid, and RPE expressed a variety of CP- and AP-related complement/complement regulatory genes [6]. Genes of the MBL pathway were only expressed at background levels [6]. In addition, our observation on cultured murine RPE cells is also similar to that of cultured human fetal RPE cells [6], with the exception of *C5*. A low level of *C5* gene expression was detected in human retina and RPE cells by real-time RT–PCR [6]. In contrast, in our study *C5* gene expression was not detected in either primary cultured mouse RPE cells or the mouse RPE cell line B6-RPE07 cells (Figure 4B) nor was it induced by cytokines IL-6, TNF-α, and INF-γ. It is possible that the discrepancy between humans and mice in relation to *C5* gene expression by RPE cells may reflect a species difference. For example, a previous study in murine brain tissue has shown that *C5* was only detectable in the cerebellum of malaria-infected but not normal mouse [29]. However, in humans *C5* was detected in several regions of the brain [30] as well as in brain oligodendroglial cells at both mRNA and protein levels [31].

In this study, we find that complement gene expression in microglia and RPE cells is regulated predominately by inflammatory cytokines IFN-γ and TNF-α and is less affected by IL-6 and LPS. Increased production of IFN-γ or TNF-α has been observed in uveoretinitis [32,33], diabetic retinopathy [34-36], and age-related macular degeneration [37]. In the normal aging retina, TNF-α gene expression is increased as compared to young retina [38]. Complement activation has been shown to be involved in the aforementioned retinal diseases [5,9-13,15-17] and in normal retinal aging [21]. Macrophages and microglial cells are the major sources of inflammatory cytokine production in the retina. Our result suggests that the two major components of the retinal innate immune system, i.e., the myeloid-derived cells and the complement system, may work together under pathophysiologic conditions. On the one hand, inflammatory cytokines produced by activated microglial cells or infiltrating macrophages may increase CP- and AP-complement gene expression, leading to retinal complement activation. On the other hand, complement activation may release complement fragments C3a and C5a, which may further affect microglia or macrophage activation and migration.

In addition to myeloid-derived cells, RPE cells also actively interact with the complement system. RPE cells express and produce a variety of complement components [6,20,21,39]. Activated RPE cells also produce various inflammatory cytokines [40-44], which may affect their own complement gene expression. Complement activation, on the other hand, may affect RPE function. For example, complement fragment C5a is able to induce inflammatory cytokines *IL-1β*, *IL-6*, *CCL2*, *IL-8* [43], and *VEGF* expression in RPE cells [45].

Therefore, retinal microglial cells, RPE cells, and the complement system, comprising the retinal innate immune system, may play an important role in retinal homeostasis. Under overt inflammatory conditions, they may interact with the adaptive immune system, contributing to retinal pathologies. Experimental autoimmune uveoretinitis, for example, is a Th1-type autoimmune disease [46,47], and the disease is characterized by macrophage and CD4 T-cell infiltration together with high levels of IFN-γ and TNF-α production [32,33]. Recent studies have shown that complement activation [11], particularly that mediated by the AP, significantly contributes to retinal pathology in experimental autoimmune uveoretinitis [38,48]. In this study, we show that IFN-γ strongly upregulates the CP- and AP-related genes (>600 fold increase in the CFB gene in microglial cells (Figure 7), suggesting that IFN-γ may play an important role in retinal complement activation in uveoretinitis.

In summary, our data suggest that the CP and AP are likely to be the main pathways involved in retinal complement activation and that RPE and retinal microglial cells contribute significantly to related complement gene expression. In addition, our findings also suggest that the tissue microenvironment, particularly the inflammatory cytokines present, may play critical roles in controlling retinal complement gene expression. However, further knowledge relating to how the retinal microenvironment may affect the expression and activation of the complement system under different pathological conditions is essential to understand the role of the complement system in different disease conditions, such as age-related macular degeneration, diabetic retinopathy, and glaucoma.
